# Central Line-Associated Bloodstream Infections in Intensive Care Unit During and After the COVID-19 Pandemic, 5-Year Prospective Observational Study

**DOI:** 10.3390/jcm14165655

**Published:** 2025-08-10

**Authors:** Jakub Sleziak, Marta Błażejewska, Wiesława Duszyńska

**Affiliations:** 1 The Students Scientific Association by Department and Clinic of Anaesthesiology and Intensive Therapy, Wroclaw Medical University, L. Pasteura Street 1, 50-367 Wroclaw, Poland; marta.blazejewska@student.umw.edu.pl; 2 Department and Clinic of Anaesthesiology and Intensive Therapy, Wroclaw Medical University, L. Pasteura Street 1, 50-367 Wroclaw, Poland; wieslawa.duszynska@umw.edu.pl

**Keywords:** CLABSI, intensive care unit, COVID-19

## Abstract

**Background/Objectives:** The COVID-19 pandemic significantly disrupted healthcare systems worldwide, leading to increased healthcare-associated infection rates, particularly in the intensive care unit (ICU) setting. Little is known about the evolution of this phenomenon in subsequent years. **Methods:** This retrospective analysis of prospectively collected data (January 2020–December 2024) examined central line-associated bloodstream infections (CLABSI) in the Wroclaw Medical University hospital’s ICU during and after the COVID-19 pandemic. **Results:** Ninety CLABSI cases were observed in 3149 ICU patients across 39,837 patient-days and 36,038 central-vascular-catheter-days (CVC-D). The mean CLABSI frequency was 2.97 per 100 admissions, with an incidence density of 2.49 per 1000 CVC-D. CLABSI occurred more frequently in males than in females (3.51% vs. 1.69%, *p* = 0.003) and in patients with concomitant SARS-CoV-2 infection than in individuals without such coinfection (6.06% vs. 1.88%, *p* = 0.00037). Microbiological analysis revealed *Staphylococcus epidermidis* as the most frequent etiological factor of CLABSI (33.3%). Alert pathogens constituted 34.26% of all CLABSI etiological factors, with higher prevalence during the pandemic than afterward (51.16% vs. 23.08%, *p* = 0.005437). Patients with CLABSI had significantly longer ICU stays (53.57 vs. 11.62 days, *p* = 0.001). After adjusting for immortal time bias using matched cohort analysis, CLABSI was not associated with increased mortality (*p* = 0.735). The overall compliance level of adherence to CLABSI prevention measures was 86.9%, with no statistically significant difference between the pandemic and post-pandemic periods, *p* = 0.417. The study did not systematically collect data on catheter types, insertion sites, or clinical circumstances (emergency vs. elective), which are known risk factors that may have influenced the observed CLABSI incidence rates. **Conclusions:** Despite increased patient volume post-pandemic, CLABSI metrics remained stable, possibly due to the successful adaptation of infection prevention protocols. However, interpretation of incidence data should consider unmeasured confounding factors. These findings address knowledge gaps regarding how the pandemic affected CLABSI epidemiology and antimicrobial resistance patterns, with implications for infection control practices during future healthcare crises.

## 1. Introduction

Hospital-acquired infections (HAIs) are a prevalent worldwide problem that worsens each year [1]. Their prevalence exhibits substantial variation, ranging from 5.7% to 19.1%, with an aggregated rate of 10.1% [2]. It was estimated that, each year, 136 million HAIs are caused by drug-resistant bacteria [3]. The intensive care units (ICUs) are among the most frequent locations for the occurrence of different clinical forms of HAIs [1]. It is estimated that around 23.1–27.6% of ICU patients suffer from HAIs and that ICU patients face the greatest risk of developing device-associated HAIs (DA-HAIs) [4,5].

Central vascular catheters (CVCs) play a crucial role in critical care settings, enabling essential interventions for patients with severe illness. These devices facilitate multiple therapeutic and diagnostic procedures, including renal replacement therapy, parenteral nutrition, hemodynamic monitoring, and administration of catecholamines, potassium, and other medications [6]. Hence, CVCs are particularly frequently used in ICU patients [7].

Despite their clinical utility, CVCs are associated with significant complications, including traumatic insertion injuries and DA-HAIs such as central line-associated bloodstream infection (CLABSI), bacteremia, endocarditis, or septic thrombophlebitis [8,9]. Among these potential complications, CLABSI represents the most clinically significant adverse outcome related to CVCs [10].

CLABSI constitutes 9.6% of HAIs in ICU patients [11]. The incidence of CLABSI is elevated among ICU patients due to frequent central line insertions and utilization of higher-risk catheter types, and emergency clinical situations frequently necessitate repeated daily access and extended duration of use [12].

The COVID-19 pandemic significantly impacted CLABSI rates, and higher prevalence of COVID-19 within hospital settings correlated with elevated CLABSI events during initial pandemic waves [13]. High workload, staff redeployment, and overwhelmed personnel likely compromised adherence to fundamental infection control measures, including proper hand hygiene protocols and compliance with central line care bundles, ultimately contributing to a significant increase in CLABSI during the pandemic [14]. The higher frequency of CLABSI during the pandemic was evident in the ICU setting [15].

CLABSI prolongs hospitalization duration, increases morbidity, and independently elevates mortality rates by 12–25% [16]. The attributable cost of a CLABSI episode was estimated at $44,000–48,000 [17,18,19]. Factors elevating CLABSI risk include chronic conditions, immunosuppression, malnutrition, age extremes, compromised skin integrity, and extended hospitalization prior to line placement [17].

Approximately 65–70% of CABSI cases may be preventable through the implementation of current evidence-based intervention strategies during catheter insertion as well as subsequent continuous catheter care [20]. This was recently emphasized when subsequent COVID-19 pandemic waves demonstrated improvements in CLABSI rates, coinciding with renewed emphasis on prevention protocols [13].

Understanding the unprecedented epidemiological shifts that occurred during and after the COVID-19 pandemic is crucial, as CLABSIs remain serious yet preventable complications. This single-center study aimed to assess infection trends while accounting for patient populations during this extraordinary period.

The three-year post-pandemic follow-up offered invaluable insights into lasting changes in CLABSI incidence, microbial etiology, and antibiotic resistance patterns that may have emerged during the crisis but continued to affect patient care long after COVID-19 cases declined. These findings address a significant knowledge gap regarding how a major global health crisis affected this specific yet critical aspect of patient safety. The literature lacks data on this problem, particularly in the Eastern European setting. By examining shifts in pathogen distribution, antimicrobial susceptibility, and clinical outcomes between pandemic and post-pandemic periods, this research may inform targeted prevention strategies, guide empiric therapy decisions, and help healthcare systems better prepare for future public health emergencies that may disrupt standard infection control practices.

## 2. Materials and Methods

### 2.1. The Study Design and Data Acquisition

This five-year prospective observational study spanning from January 2020 to December 2024 examined CLABSI epidemiology at the Department of Anesthesiology and Intensive Therapy of Wroclaw Medical University. The investigation incorporated 3149 ICU admissions with monthly ICU infection reports from the Infection Monitoring and Treatment Laboratory infection surveillance, annual microbiological reports, and the hospital database.

Daily documentation on standardized surveillance instruments facilitated the quantification of the number of treated patients, patient-days (Pt-D), CVC-D, and derivation of the central vascular catheter utilization ratio (CVC-UR). The investigation’s principal objective was to establish the CLABSI incidence among ICU patients. Patient demographic data were stratified by gender and the recent clinical history precipitating ICU admission (classified as medical or surgical). CLABSI surveillance adhered to standardized indicators established by the European Centre for Disease Prevention and Control (ECDC) protocol [21]. The investigation examined microbiological factors associated with these infections and evaluated adherence to CAUTI prevention protocol elements as specified by the Centers for Disease Control and Prevention’s (CDC) National Healthcare Safety Network (NHSN) guidelines [22]. These compliance data were collected on a biweekly basis by trained student researchers from the Students’ Science Club throughout the period spanning January 2020 to December 2024. A retrospective analysis of prospectively collected data was conducted with a focus on comparison of the pandemic (2020–2021) and three consecutive years, 2022–2023, referred to as the “post-pandemic” period.

### 2.2. CLABSI Diagnostic Criteria

The CLABSI diagnosis methodology employed in this study required a two-tiered assessment approach. Initially, bloodstream infection (BSI) was established through either a single positive blood culture yielding a recognized pathogen or through the presence of clinical manifestations (fever > 38°C, chills, or hypotension) coupled with two positive blood cultures for common skin contaminants (coagulase-negative staphylococci, *Micrococcus* spp., *Propionibacterium acnes*, *Bacillus* spp., or *Corynebacterium* spp.) obtained from separate samples within a 48 h window. Subsequently, central line association was determined when the BSI occurred >48 h after central catheter placement or within 48 h after catheter removal and demonstrated microbiological concordance with one of the following: quantitative CVC culture yielding ≥10^3^ CFU/mL or semi-quantitative CVC culture exceeding 15 CFU; differential time to positivity with CVC samples becoming positive at least two hours before peripheral samples were drawn simultaneously, and identical microorganism isolation from the blood and the catheter [21]. A CVC, also referred to as a central line, was defined as an intravascular catheter with its terminal end positioned within one of the major vessels. Within the NHSN surveillance protocol for CLABSI reporting and central line utilization quantification, the following are classified as major vessels: aorta, pulmonary artery, superior and inferior venae cavae, brachiocephalic veins, internal jugular veins, subclavian veins, external and common iliac veins, and common femoral veins [19]. It is important to note that the CLABSI surveillance definition serves primarily as a monitoring and reporting tool rather than as clinical diagnostic criteria, as it demonstrates reduced specificity for patient-level diagnosis [23]. Patients were excluded from the study if they presented with CLABSI diagnosed upon admission to the ICU or if their CLABSI was attributed to vascular ports or other permanent catheters that had been implanted prior to their ICU admission.

### 2.3. Microbiological Analysis and Pathogen Identification

Microbiological analyses were conducted at the Microbiological Laboratory of the University Hospital in Wrocław. Pathogen identification, antimicrobial susceptibility profiling, and resistance mechanism characterization were performed in accordance with European Committee on Antimicrobial Susceptibility Testing (EUCAST) guidelines and standardized European/Polish microbiological diagnostic protocols [24]. The identification and stratification of multidrug-resistant bacterial isolates designated as “alert pathogens” adhered to the classification system established by Magiorakos et al. [25]. The alert pathogen classification encompassed the following: Gram-negative organisms (including Enterobacteriaceae, *Pseudomonas aeruginosa*, and *Acinetobacter* spp.) exhibiting multidrug-resistant (MDR) or extensively drug-resistant (XDR) phenotypes and Gram-positive alert pathogens including methicillin-resistant *Staphylococcus aureus* (MRSA) and glycopeptide-resistant enterococci (GRE).

### 2.4. Surveillance Metrics and Epidemiological Parameters

The epidemiological indicators of CLABSI were quantified using the following formulae: CLABSI incidence density was calculated as the quotient of the number of CLABSI cases divided by the total number of CVC-D, multiplied by 1000. The CLABSI incidence rate was determined by dividing the number of CLABSI cases by the total number of Pt-D, multiplied by 1000. CLABSI frequency was calculated as the number of CLABSI cases per 100 patients admitted to the ICU over a time interval, multiplied by 100. The CVC-UR was calculated as the proportion of central CVC-D to Pt-D presented as a percentage. Adherence to CLABSI prevention bundles as specified in CDC guidelines was assessed by calculating the percentage of successfully implemented bundle components relative to the total number of observations conducted.

### 2.5. Ethical Approval

Patient-related information and microbiological testing data were systematically collected within the framework of standard clinical care protocols and infection surveillance procedures. Throughout data acquisition and manuscript development, patient confidentiality was rigorously preserved in accordance with established ethical standards. Per the regulatory framework of the Bioethics Committee of Wroclaw Medical University, explicit documentation of informed consent and written patient declarations were deemed unnecessary for this investigational protocol. The study received ethical approval under reference identification KB-576/2016 issued on 21 December 2016.

### 2.6. Statistical Analysis

Statistical analyses were performed utilizing the Microsoft^®^ Excel (Version 16.89.1) Data Analysis ToolPack. Categorical variables were expressed as frequencies and percentages, whereas continuous variables were presented as mean ± standard deviation (SD), median ± interquartile range (IQR), or with 95% confidence intervals (CI). Inter-group comparisons of categorical variables were conducted using the chi-square test or Pearson’s chi-square test with Yates’ correction, as methodologically appropriate. Analysis of continuous variables was executed via the two-tailed Student’s t-test or single-factor analysis of variance (ANOVA), contingent upon the number of groups under comparison. For correlation analyses, Pearson’s correlation coefficients were computed, followed by t-statistic calculation and subsequent conversion to probability values using Student’s t-distribution with n-2 degrees of freedom. Kaplan–Meier curves were constructed to estimate survival probabilities from the index date (day of CLABSI diagnosis for cases, corresponding hospitalization day for matched controls), with between-group differences assessed using the Cox–Mantel log-rank test. Statistical significance was established at *p* < 0.05.

## 3. Results

### 3.1. Patient Demographics and Study Population

Over the five-year observational period, a total of 3149 patients were admitted to the ICU, consisting of 2022 (64.2%) males and 1127 (35.8%) females. The majority were surgical patients (2194; 69.7%), while patients with internal-medicine conditions constituted 955 (30.3%) of the cohort. Throughout 39,837 patient-days (Pt-D) and 36,038 CVC-D, the mean CVC utilization ratio (CVC-UR) was 90.46%. Table 1 summarizes the annual characteristics of department patients along with Pt-D, CVC-D, and CVC-UR (Table 1).

### 3.2. CLABSI Incidence and Epidemiological Indicators

A total of 90 CLABSI cases were diagnosed in 87 patients throughout the study period. The overall CLABSI frequency was 2.97 per 100 admissions (CI95%: 2.25–3.7), with an incidence rate of 2.28 per 1000 Pt-D (CI95%: 1.73–2.84) and an incidence density of 2.49 per 1000 CVC-D (CI95%: 1.9–3.07). Table 2 summarizes the annual epidemiological indicators of CLABSI (Table 2).

### 3.3. Gender and Patient Type Distribution

CLABSI occurred significantly more frequently in males (71/2022; 3.51%) than in females (19/1127; 1.69%), *p* = 0.003. Among CLABSI cases, 71 (78.9%) were male and 19 (21.1%) were female. The distribution between surgical (58/2194; 2.64%) and medical (32/955; 3.35%) patients showed no significant difference, *p* = 0.253. Table 3 details the annual distribution of CLABSI cases by gender and patient type (Table 3).

### 3.4. Mortality Analysis

The overall mortality among patients with CLABSI was 20% (18/90). No statistical significance was observed between the mortality of males, 16/71 (22.54%), and females, 2/19 (10.53%), with CLABSI, *p* = 0.245. However, medical patients with CLABSI experienced significantly higher mortality (11/32; 34.38%) compared to surgical patients (7/58; 12.07%), *p* = 0.0138. Unexpectedly, patients with CLABSI exhibited lower mortality than those without CLABSI, 18/90 (20%) vs. 957/3059 (31.28%), *p* = 0.022. No statistically significant difference was observed between the mortality of CLABSI caused by alert pathogens 8/30 (26.67%) compared to non-alert pathogens 10/60 (16.67%), *p* = 0.335.

#### Matched Cohort Analysis

To address potential immortal time bias in comparing mortality between patients with and without CLABSI, we conducted a matched cohort analysis. From our cohort, 76 patients who developed CLABSI were initially eligible for analysis based on characteristics comparable to the control group.

The matching protocol was implemented as follows: for each CLABSI patient, we calculated the time interval (t) between ICU admission and CLABSI diagnosis. Subsequently, we randomly selected up to two control patients who (1) matched the index patient for sex, clinical characteristics, SARS-CoV-2 coinfection status, and hospitalization period, (2) did not develop CLABSI during their entire ICU stay, and (3) had a minimum length of stay ≥ t days, ensuring they remained at risk for the entire pre-CLABSI exposure period of their matched case.

For the survival analysis, the observation period began on the day of CLABSI diagnosis for index patients (defining this as day 0). For matched controls, the observation period began on day t of their ICU stay (also defined as day 0 for analysis), corresponding to the pre-CLABSI duration of their matched case. This design ensures that both CLABSI patients and their matched controls had equivalent exposure time before the start of observation, thereby eliminating immortal time bias.

Due to these stringent matching criteria requiring adequate length of stay in controls, only 147 control patients met eligibility requirements from the intended 152 matches (2:1 ratio).

**Figure 1:**
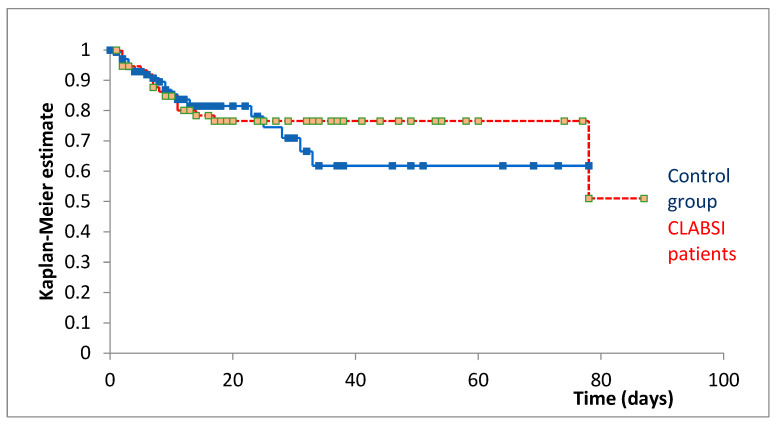
Kaplan–Meier survival curves comparing patients with CLABSI (red, *n* = 76) and matched controls (blue, *n* = 147) from the index date. The index date represents the day of CLABSI diagnosis for infected patients and the corresponding day of hospitalization (day t) for matched controls.

Kaplan–Meier survival analysis from the index date revealed comparable survival trajectories between groups (Figure 1). At 20 days post-index date, survival was 81.49% for patients without CLABSI and 76.58% for patients with CLABSI.

The Cox–Mantel log-rank test showed no statistically significant difference in survival between patients with and without CLABSI (*p* = 0.735), indicating no significant association between CLABSI development and mortality when accounting for immortal time bias through appropriate matching and index date alignment.

### 3.5. Length of Stay Analysis

The average duration of hospitalization at the ICU-LOS in the total period equaled 12.76 days, CI95% (12.13–13.39), median 7 (IQR: 4–14). Patients with CLABSI had substantially longer stays (mean: 53.57 days, CI95%: 41.39–65.75) compared to those without infection (mean: 11.62 days, CI95%: 11.12–12.12), *p* = 0.001.

CLABSI typically occurred on the 19th day of hospitalization (median; IQR: 14–31.75). No significant differences were found in the timing of CLABSI occurrence between alert pathogen (29.03 days, CI95%: 19.88–38.18) and non-alert pathogen infections (33.35 days, CI95%: 22.52–44.18), *p* = 0.54. Similarly, LOS did not differ significantly between patients with alert pathogen CLABSI (52.8 days, CI95%: 36.55–69.05) and non-alert pathogen CLABSI (62.32 days, CI95%: 44.64–80), *p* = 0.425.

Notably, the mean LOS in 2024 (10.91 days) was significantly shorter than in previous years (13.33 days), *p* = 0.001.

### 3.6. Microbiological Analysis

**Figure 2:**
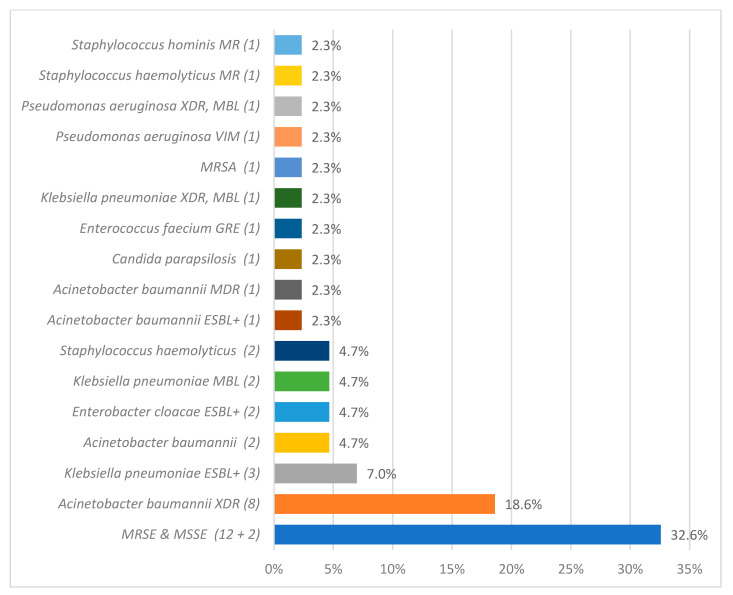
The overall count of specific etiological agents responsible for central line-associated bloodstream infection (CLABSI), as identified through microbiological analysis, during the pandemic period. methicillin-resistant (MR), extended-spectrum beta-lactamase (ESBL), glycopeptide-resistant enterococci (GRE), methicillin-resistant *Staphylococcus aureus* (MRSA), extended-spectrum beta-lactamase positive (ESBL+), extensively drug-resistant (XDR), New Delhi metallo-beta-lactamase (NDM), and methicillin-resistant *Staphylococcus epidermidis* and methicillin-susceptible *Staphylococcus epidermidis* (MRSE and MSSE, respectively).

**Figure 3:**
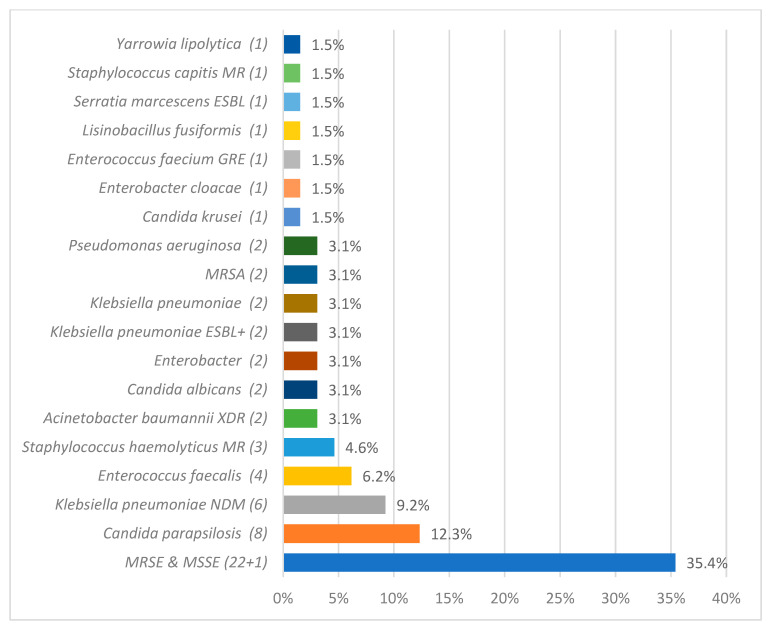
The overall count of specific etiological agents responsible for central line-associated bloodstream infection (CLABSI), as identified through microbiological analysis, after the pandemic period. methicillin-resistant (MR), extended-spectrum beta-lactamase (ESBL), glycopeptide-resistant enterococci (GRE), methicillin-resistant *Staphylococcus aureus* (MRSA), extended-spectrum beta-lactamase positive (ESBL+), extensively drug-resistant (XDR), New Delhi metallo-beta-lactamase (NDM), and methicillin-resistant *Staphylococcus epidermidis* and methicillin-susceptible *Staphylococcus epidermidis* (MRSE and MSSE, respectively).

Microbiological analysis identified 108 pathogens as CLABSI etiological factors. The most frequently isolated pathogens from blood samples were *Staphylococcus epidermidis* 37 (34.26%)*, Klebsiella pneumoniae* 16 (14.81%*), Acinetobacter baumannii* 14 (12.96%), and *Candida parapsilosis* 9 (8.33%). The detailed data regarding CLABSI etiology during and after the COVID-19 pandemic are described in Figure 2 and Figure 3.

### 3.7. Antimicrobial Resistance Patterns

The alert pathogens comprised 37/108 (34.26%) of all CLABSI etiological factors. Methicillin resistance was the predominant antimicrobial resistance (AMR) mechanism affecting 43/108 (38.81%) of isolated pathogens. The ESBL producers accounted for 9/108 (8.33%), and 11/108 (10.19%) of pathogens were MBL producers. In addition, 12/108 (11.11%) of pathogens were classified as XDR.

**Figure 4:**
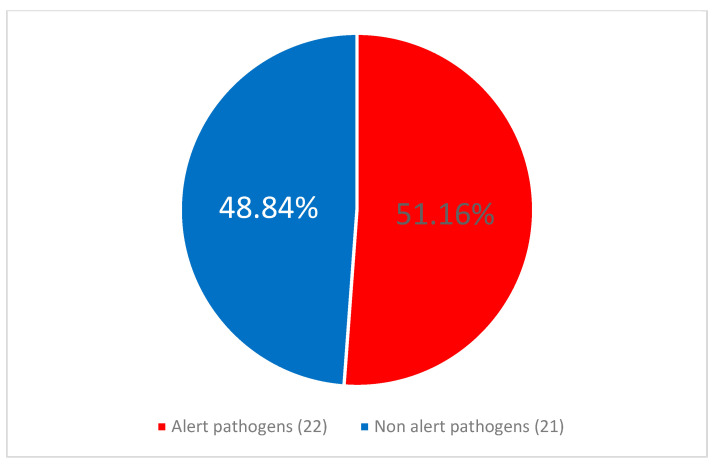
The proportion of alert pathogens among the central line-associated bloodstream infection (CLABSI) etiological agents during the pandemic period.

**Figure 5:**
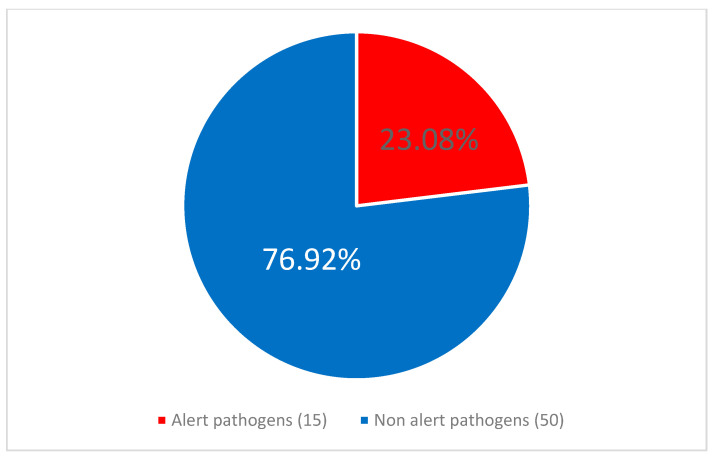
The proportion of alert pathogens among the central line-associated bloodstream infection (CLABSI) etiological agents after the pandemic period.

No statistically significant difference was observed between the frequency of alert pathogens as etiology of CLABSI in COVID-infected 7/16 (43.75%) vs. patients without COVID-19 coinfection, 10/17 (58.82%), *p* = 0.674. CLABSI was significantly more frequently caused by alert pathogens during 17/33 (51.52%) than after 13/57 (22.81%) of the pandemic, *p* = 0.0047. Similarly alert pathogens were identified as etiological factors of CLABSI more frequently during than after the pandemic, *p* = 0.005. This relationship is illustrated in Figure 4 and Figure 5.

### 3.8. Pandemic and Post-Pandemic Periods Comparison

The post-pandemic period demonstrated increased healthcare utilization, with higher mean monthly Pt-D and CVC-D numbers. Despite this increased volume, CLABSI epidemiological indicators remained stable between periods, with no significant differences in CLABSI frequency and density. The levels of CVC-UR remained at a constant level. Table 4 presents the comprehensive comparison of these metrics (Table 4).

During the pandemic, CLABSI occurred more frequently in patients with concomitant SARS-CoV-2 coinfection in comparison to patients without such coinfection.

No statistically significant difference was found between CLABSI mortality during (5/33, 15.15%) and after the pandemic (13/57, 22.81%), *p* = 0.553. Table 5 summarizes key CLABSI characteristics in relation to COVID-19 coinfection status.

### 3.9. CLABSI Preventive Measures Assessment

Analysis of 1342 CLABSI care bundle protocols [22] revealed an overall compliance rate of 86.9% (CI95%: 90–100%). The most consistently fulfilled criterium was the sterile dressing presence, 98.6% (97.74–100%), while chlorhexidine-impregnated dressing use was the least common 34.5% (25.79–42.33%).

Comparing pandemic and post-pandemic periods revealed no significant difference in overall compliance (85.5% vs. 88.48%, *p* = 0.417). However, the hand hygiene regime before the manipulation of CVC was significantly more strictly obeyed during the pandemic. Dressings impregnated with chlorhexidine were applied more frequently after than during the pandemic. Table 6 details the adherence to individual prevention criteria.

The strongest negative correlation between the compliance rate and CLABSI incidence density equaled −0.45 and was observed for chlorhexidine-impregnated dressing usage; however, no statistical significance for such correlation was observed for this, *p* = 0.984, nor for any other criterium or for total adherence level, *p* = 0.93.

## 4. Discussion

Our study aimed to evaluate CLABSI in the ICU setting comprehensively, focusing on infection characteristics, incidence rates, and microbiological profiles along with antibiotic resistance patterns. This approach was designed to provide a thorough understanding of CLABSI dynamics in critical-care environments using a multifaceted methodology. A better understanding of observations is enabled through contextualization of findings within the broader perspective of previous research and international literature.

The assessment of CLABSI frequency based on epidemiological indicators was the first issue analyzed in this research. The incidence of CLABSIs varies globally, influenced by regional microbial flora, infection control practices, and factors such as healthcare infrastructure [26]. Trend analysis of yearly median incidence density in ICUs from five European countries demonstrated a change for CLABSIs from the previous decreasing trend since 2008 to a sharp increase in 2020, with a small decrease in 2021 [27]. The ECDC’s Annual Epidemiological Report (AER) of HAI in European ICUs for 2020 found a mean CLABSI incidence density of 4.9 (median: 2.5, IQR: 0–7.6). The CVC-UR was 80.90% [26]. The 2021 ECDC AER showed a mean CLABSI incidence density of 4.1 episodes per 1000 CVC-D (median: 3.2, IQR: 1.0–6.0) [27]. The CVC-UR remained at exactly the same level as in the previous year. The increased trends of CLABSI in 2020 with a slight decrease in 2021 observed in the ECDC’s reports mirror the pattern observed in our unit; nevertheless, the incidence densities from the corresponding years were lower in our department, respectively 3.09, IQR (1.43–4.4) and 2.11, IQR (0–3.73).

A relationship similar to European trends was observed in the USA, where CLABSI incidence levels were continuously declining and dropped by 31% from 2015 to 2019 [28]. Then, an early pandemic report from the NHSN found significantly increased counts of CLABSIs during the early months of the pandemic, with the ICUs marked by the greatest increase (39%) from 0.75 in 2019 to 1.04 in 2020 [29].

Across the USA’s ICUs, the mean pandemic CLABSI incidence density, 1.23 CI95% (1.15–1.3), was significantly higher than in the post-pandemic years (2022–2023), 0.99 CI95% (0.91–1.07), *p* = 0.00003. Moreover, the mean incidence density of CLABSI in years 2020–2023, 1.11 CI95% (1.05–1.16), was significantly lower in comparison to that observed in our unit in the corresponding time interval, 2.6 CI95% (1.95–3.24), *p* = 0.00003 [30].

Data from seven low- and middle-income countries collected by INICC indicate that CLABSI rates increased substantially, rising from 2.54 to 4.73 infections per 1000 CVC-D between 2019 and 2020, coinciding with the COVID-19 pandemic [31].

From the long-term perspective of 24 years of observation performed by INICC in 41 countries from Asia, Eastern Europe, Latin America, and the Middle East, the mean CLABSI rate was established at 4.82 cases per 1000 CVC-D, which is almost twice the density observed in our department in the total period [32].

The findings of this study were further compared to the findings of similar studies from the same department in the past. In the years 2007–2010, the mean CLABSI incidence density reached 4.01, CI95% (2.8–5.6) [33]. Then, in the year 2012, it was 3.77, and one year later, it was estimated at 3.36 [34]. In the years 2015–2017, the CLABSI incidence density was 1.63 (IQR: 1.47–2.09), around 35% less than in the total observed period from our study [11]. It becomes apparent that in our department, the COVID-19 pandemic brought an abrupt increase in CLABSI incidence that was gradually declining over the previous 10 years and that this change persisted through the following three years.

This finding likely stems from multiple interrelated factors. The unprecedented strain on ICU resources during the pandemic necessitated rapid modifications to standard infection prevention protocols, with healthcare workers facing increased workloads, potential protective equipment shortages, and modified care routines designed to minimize exposure risk [35,36]. These emergency adaptations have unintentionally compromised adherence to established central line insertion and maintenance bundles [37]. This potentially created conditions where lines remained in place longer due to challenges in performing timely removal assessments. The continued elevation in CLABSI rates post-pandemic suggests that these disruptions may have led to more sustained changes in clinical practice patterns or institutional memory loss regarding best practices at the unit. Such changes may have been difficult to reverse despite the resumption of normal operations.

The mean CLABSI incidence density in the years 2007–2016 in another Polish hospital was found to be much higher and reached 8.0 [4]. This may indicate substantial room for improvement in HAI prevention strategies across healthcare institutions in Poland. Further multicenter studies are needed to identify the specific factors contributing to this variability and to develop national strategies that can help reduce CLABSI rates uniformly throughout the country’s healthcare system.

The emergence of COVID-19 correlated with notable increases in HAI across healthcare settings, stemming from multiple factors including elevated hospitalization volumes, staffing shortages, and delayed access to care. Statistical analyses demonstrated significantly higher CLABSI rates during the COVID-19 period compared to pre-pandemic baselines, with a direct correlation between CLABSI incidence and COVID-19-related hospitalizations in both ICU and acute care environments [38].

The difference between the greater crude incidence density of CLABSI in our department during the pandemic in comparison to post-pandemic levels did not achieve statistical significance. Nevertheless, data from the USA’s ICUs confirm such a difference with statistical significance [30]. Interestingly, some ICUs noted decreased CLABSI incidence rates during the pandemic [39].

In our study, males developed CLABSI more frequently than females. This finding aligns with the results of other studies on this topic [40,41]. Furthermore, we found a significantly greater CLABSI incidence in patients with SARS-CoV-2 coinfection. Findings of corresponding studies confirm this relationship. A retrospective study carried out in multiple ICUs in Saudi Arabia found significantly greater CLASBI incidence densities among COVID-19 patients in comparison to patients without such coinfection (6.18 vs. 3.77, *p* = 0.006) [42]. A prospective multicentric study found a greater risk of BSI incidence in COVID-19-infected ICU patients (14.9% vs. 3.4%, *p* < 0.0001) [43]. The authors indicated that this increased risk was associated with tocilizumab or anakinra treatment.

In our research, no statistically significant difference was observed between the mortality of CLABSI during the pandemic in comparison to the subsequent years. The data in the literature regarding this topic are scarce. The study from Saudi Arabia showed that the mortality of CLABSI was greater during the pandemic than before (1.79 vs. 0.93, *p* = 0.003); however, no data regarding the post-pandemic period were included [42].

Interestingly, our study found greater mortality in patients without CLABSI. This could be attributed to the fact that the average time from admission to death was established at 10 days CI95% (9.21–10.78), median 6, and the average day of hospitalization when CLABSI was diagnosed was 32.92 CI95% (23.45–42.39), median 19. Therefore, one can assume that the higher mortality in the group of patients without CLABSI may stem from the fact that patients in severely compromised conditions may have died before developing CLABSI. Indeed, the time-dependent bias was recognized in a previous meta-analysis on this topic [44], and in fact, propensity toward CLABSI, such as a high Acute Physiology and Chronic Health Evaluation (APACHE) II score, was found to be associated with mortality more profoundly than the infection itself [45].

To address this time-dependent bias, we performed a matched cohort analysis with aligned observation periods starting from CLABSI diagnosis (or corresponding hospitalization day for controls), which revealed no significant mortality difference between groups, confirming that the apparent survival disadvantage in non-CLABSI patients was indeed an artifact.

The literature delivers numerous proofs for an association between CLABSI and increased mortality rates [46]. Some authors argue that, while CLABSIs independently increase both healthcare costs and duration of hospitalization, they may not independently contribute to increased mortality when controlling for other factors [47]; however, the latter was disproved by a large meta-analysis including mainly ICU-based studies [46].

Our study found no statistically significant increase in mortality in CLABSI caused by alert pathogens. This state is in opposition to findings of a case-control study conducted in Cali, Colombia, over a four-year period, where patients who had CLABSI with resistant microorganisms experienced significantly higher mortality odds, 4.04 times greater (CI95% 1.17–13.96, *p* = 0.027), compared to patients whose CLABSIs were caused by antibiotic-sensitive organisms [48]. Another study conducted in an ICU in São Paulo, Brazil, 2016–2020, documented substantially higher hospital mortality rates among patients with MDR CLABSIs (57.6% versus 30.3%, respectively). Nevertheless, these single-center studies did not match our project for the time interval and the facility type.

In our study, the mean LOS of patients with CLABSI was around 42 days longer than the mean LOS of patients without such infection. This observation may represent a bidirectional relationship wherein CLABSI potentially extends hospitalization duration, while simultaneously extended central venous catheterization periods associated with longer hospitalizations increase CLABSI incidence probability. Literature indicates that CLABSI causes an excess length of stay of 24 days in ICU patients [33] and that CLABSI risk increases along with the time of presence of central line, and length of hospitalization [32,41,49].

The microbiological profile of HAIs worldwide varies significantly [1]. Regarding the etiology of ICU-acquired BSIs in the ECDC’s AERs, coagulase-negative staphylococci were the most frequently isolated microorganisms in both 2020 (26.7%) and 2021 (24.9%), followed by *Enterococcus* spp. (18.9% in 2020; 19.1% in 2021) and *Klebsiella* spp. (11.5% in 2020; 11.3% in 2021) [26,27]. This remains in accordance with our findings since, at our department, MRSE was the most frequent CLABSI etiological factor in those years.

The microbiological profile of CLABSI in our unit evolved over time. In the years 2012–2014, the main etiological factors of CLABSI were Enterobacteriaceae (29%), coagulase-negative staphylococci (22%), and *Staphylococcus aureus* (21%) [34]. In the years 2015–2017, the predominant etiological agents of CLABSI were methicillin-resistant coagulase-negative staphylococci [11].

The high percentage of alert pathogens among CLABSI etiological factors may be attributed to several factors. Infection control practices may have been potentially compromised with laboratories burdened with COVID-19 testing indirectly interrupting the detection of these bacteria [50]. The empiric use of broad-spectrum antibiotics for COVID-19 patients with suspected bacterial co-infections likely created selective pressure favoring resistant organisms [51].

The post-pandemic reduction in alert pathogen prevalence in our study suggests the potential reversibility of these concerning trends with the restoration of robust antimicrobial stewardship and infection prevention practices. This underscores the importance of maintaining vigilant antimicrobial stewardship during future healthcare crises to prevent further acceleration of AMR.

The higher prevalence of alert pathogens as etiological factors during the pandemic was similarly found in other studies [14]. While many established CLABSI risk factors, such as country income, hospitalization, or ICU type, cannot be altered, reduction in LOS, CVC-D, and adherence to CLABSI preventing recommendations are feasible options for reducing the infection’s frequency [32].

The nurses play the key role in HAIs and, therefore, CLABSI prevention [52]. In New York in the initial phase of the COVID-19 pandemic, the personnel had difficulties with maintaining the proper adherence to the CLABSI prevention protocols, which was reflected in increased CLABSI frequencies, which was later mitigated by introduction of qualified infusion nurses that by performing daily maintenance bundle rounds and educating co-workers contributed to a significant decrease in this infection [37].

According to a recent survey, ICU nurses in Poland have either good or very good knowledge of CLABSI prevention, but standardized guidelines and continuous training are still needed for improved outcomes [53].

The comparison of hospitalization parameters reveals a significant shift in healthcare utilization patterns following the pandemic period. Despite handling substantially more patients and providing more intensive care services post-pandemic, infection control effectiveness appears to have remained resilient, as evidenced by the stability of CLABSI metrics across both periods. This suggests successful adaptation of the infection prevention protocol. The consistency in the CVC utilization ratio throughout both periods indicates that central line insertion practices remained standardized regardless of pandemic-related disruptions, potentially contributing to the stability in infection rates despite increased patient volume.

This prospective observational study was designed with predefined protocols and diagnostic criteria established before data collection (which began in January 2020). While the COVID-19 pandemic emerged during the observation period, our systematic approach to CLABSI surveillance remained consistent, with the addition of SARS-CoV-2 infection status as a monitored parameter to capture this unprecedented influence on ICU epidemiology. The temporal boundaries of our study (January 2020 to December 2021 for the pandemic period) were defined based on global and local public health declarations and healthcare system impacts. While these boundaries may not perfectly capture all pandemic-related effects, they align with internationally recognized periods of maximum ICU burden and allow for meaningful comparisons with other studies.

This study had several important limitations that should be considered when interpreting the results. First, our data collection methods did not capture information regarding catheter types, despite evidence that CLABSI rates vary significantly by catheter type [54]. Second, our study did not assess several well-established risk factors for CLABSI. We did not collect detailed data on catheter insertion sites, despite evidence that femoral central venous catheters (CVCs) carry the highest CLABSI risk, followed by internal jugular and subclavian approaches [17]. Similarly, we lacked information on insertion circumstances (emergent vs. elective), catheter type, use of total parenteral nutrition, ongoing chemotherapy treatment, immunosuppression status, and operator proficiency [46]. The absence of this data limits our ability to fully contextualize the observed incidence rates and may mask important variations in infection risk across different clinical scenarios.

Nevertheless, the data stemming from analysis of CLABSI prevention bundles when extrapolated on the total period indicate that the main site for CVC placement in our department was the internal jugular vein (70.64%), followed by subclavian (21.68%) and femoral (7.68%) locations, and that the most commonly used CVCs had three lumens (99.03%).

Additionally, our study did not collect data on illness severity scores such as the APACHE II or sequential organ failure assessment score at ICU admission or at the time of CLABSI diagnosis. These severity indicators are important prognostic tools that could have provided context for interpreting mortality outcomes and allowed for risk adjustment when comparing patient groups [45]. The absence of these scores limits our ability to determine whether differences in baseline illness severity contributed to the observed outcomes.

Furthermore, we did not systematically collect data on several important clinical variables that may influence CLABSI risk and outcomes. Specifically, we lack information on the number of patients with immunosuppression or those receiving antimicrobial therapy at ICU admission and at CLABSI diagnosis, the development of septic shock at or after CLABSI diagnosis, the incidence of acute renal failure requiring renal replacement therapy, and the occurrence of multiorgan failure. Their absence from our dataset represents an important limitation that may have influenced our findings, particularly regarding the unexpected mortality patterns observed.

The lack of these clinical severity and comorbidity data should be considered when interpreting our results.

Third, due to safety reasons, the data regarding CLABSI bundle protocol realization were not collected from the SARS-CoV-2-infected patients who remained in isolation during the pandemic.

Fourth, as a single-center study, our findings may not be generalizable to other healthcare settings with different patient populations, infection control practices, or resource availability.

Furthermore, the statistical power of our study was limited for certain subgroup analyses, particularly for COVID-19-positive CLABSI cases (*n* = 16) and comparisons between alert and non-alert pathogens. While our overall sample size of 90 CLABSI cases over 5 years provides adequate power for primary analyses, readers should interpret subgroup findings with caution, recognizing that smaller sample sizes may have limited our ability to detect clinically meaningful differences.

Our matched cohort analysis has several limitations. First, while effectively addressing immortal time bias, this design excludes patients who died or were discharged in a time shorter than the corresponding intervals between admissions and CLABSI onsets, potentially limiting generalizability to the most critically ill patients.

Second, the requirement for control patients to have hospitalization durations at least equal to the pre-CLABSI period of matched cases resulted in incomplete matching (147 of 164 intended controls), which may have reduced statistical power and biased the control group toward patients with longer stays.

Third, unmeasured confounders such as the timing of central line placement, adherence to CLABSI prevention bundles, or subtle differences in illness severity could influence both CLABSI risk and mortality outcomes.

Fourth, the log-rank test used in our survival analysis may have reduced statistical power to detect differences between groups due to the crossing of the Kaplan–Meier curves, which violates the proportional hazards assumption underlying this test.

Finally, by conditioning on survival to the index date, our analysis examines whether CLABSI affects subsequent survival among patients who survive long enough to be at risk, rather than the overall impact of CLABSI on ICU mortality from admission.

Future research on CLABSI epidemiology should expand beyond single-center studies to establish multi-center collaborations across diverse geographic regions, enhancing the generalizability and validation of findings. Researchers should investigate which CLABSI prevention strategies demonstrate resilience during healthcare crises, with particular emphasis on maintaining bundle compliance under extreme system stress. Deeper microbiological and molecular investigations are needed to understand the mechanisms driving pathogen shifts and AMR development observed during the pandemic. Economic impact analyses would help quantify both direct healthcare costs and indirect societal burdens associated with pandemic-related changes in CLABSI patterns. Extended longitudinal surveillance beyond five years would determine whether the observed changes in pathogen ecology and resistance patterns represent permanent shifts or temporary disruptions. Furthermore, future projects on similar problematics should collect detailed data on illness severity scores and clinical variables described above in the limitations outlined in Section 4. Additionally, incorporating healthcare worker perspectives through mixed-methods research could identify barriers to CLABSI prevention during crises and inform more effective protocols and training programs for future emergency preparedness.

## 5. Conclusions

This five-year prospective observational study provides valuable insights into CLABSI dynamics in the ICU setting during and after the COVID-19 pandemic. Our findings demonstrate that while CLABSI incidence remained relatively stable throughout the study period, significant shifts occurred in the microbial profile. The pandemic period was marked by a higher prevalence of CLABSI in COVID-19 patients. The concerning prevalence of alert pathogens was significantly higher during the pandemic, suggesting a heightened AMR burden during the crisis. Despite increased patient volume and care intensity in post-pandemic years, infection control effectiveness remained resilient, as evidenced by stable CLABSI rates. The substantially longer LOS (53.57 vs. 11.62 days, *p* = 0.001) underscores the bidirectional relationship between risk factors and consequences of this infection. This study addresses critical knowledge gaps regarding how major healthcare disruptions affect specific aspects of patient safety, with particular relevance to Eastern European settings where data are limited. These findings can inform targeted prevention strategies, guide empiric therapy decisions, and help healthcare systems better prepare for future public health emergencies that may compromise standard infection control practices.

## Figures and Tables

**Table 1:** The characteristics of patients for each year are presented along with the percentage of the total number of patients in that year. The number of patient-days (Pt-D), central-vascular-catheter-days (CVC-D), and the central vascular catheter utilization ratio (CVC-UR) for each specific year, *n* (% of the total).

Category	2020	2021	2022	2023	2024
No. of patients	570 (100%)	598 (100%)	572 (100%)	643 (100%)	766 (100%)
Male	360 (63.2%)	372 (62.2%)	367 (64.2%)	410 (63.8%)	513 (67.0%)
Female	210 (36.8%)	226 (37.8%)	205 (35.8%)	233 (36.2%)	253 (33.0%)
Medical patients	231 (40.5%)	256 (42.8%)	141 (24.7%)	140 (21.8%)	187 (24.4%)
Surgical patients	339 (59.5%)	342 (57.2%)	431 (75.3%)	503 (78.2%)	579 (75.6%)
Pt-D	6517	7553	8704	8154	8909
CVC-D	5912	6804	7925	7354	8043
CVC-UR	90.72%	90.08%	91.05%	90.19%	90.28%

**Table 2:** Mean annual values of three epidemiological indicators (frequency, rate, and density) of central line-associated bloodstream infection (CLABSI) presented with a 95% confidence interval for those factors presented in the parentheses. The calculations for those values are described in Section 2.4.

CLABSI Epidemiological Indicators	2020	2021	2022	2023	2024
CLABSI frequency	3.14 (1.58–4.69)	2.53 (1.08–3.99)	4.05 (2.24–5.85)	2.86 (0.48–5.23)	2.28 (0.57–4)
CLABSI rate	2.8 (1.45–4.14)	1.9 (0.72–3.09)	2.55 (1.54–3.55)	2.26 (0.49–4.03)	1.91 (0.37–3.45)
CLABSI density	3.09 (1.63–4.54)	2.11 (0.84–3.39)	2.8 (1.7–3.89)	2.39 (0.58–4.2)	2.05 (0.45–3.65)

**Table 3:** Number of central line-associated bloodstream infection (CLABSI) cases recorded during the examined period annually categorized by gender (male and female) and patient type (medical and surgical), *n* (% of the total).

CLABSI	2020	2021	2022	2023	2024
TOTAL	18	15	22	18	17
In men	13 (72.2%)	14 (93.3%)	15 (68.2%)	16 (88.9%)	13 (76.5%)
In women	5 (27.8%)	1 (6.7%)	7 (31.8%)	2 (11.1%)	4 (23.5%)
In medical patients	9 (50%)	10 (66.7%)	5 (22.7%)	4 (22.2%)	4 (23.5%)
In surgical patients	9 (50%)	5 (33.3%)	17 (77.3%)	14 (77.8%)	13 (76.5%)

**Table 4:** Hospitalization trends and central line-associated bloodstream infection (CLABSI) metrics during the pandemic (2020–2021) and post-pandemic (2022–2024) periods, including sample size (*n*) and 95% confidence interval.

Parameter	Pandemic Period	Post-Pandemic Period	*p*-Value
No. of hospitalized patients [mean monthly]	1168 [48.67 (44.93–52.4)]	1981 [54.97 (50.81–59.14)]	0.034
Mean monthly Pt-D	586.25 (545.23–627.27)	715.75 (700.04–731.46)	0.000001
Mean monthly CVC-D	529.83 (489.8–569.86)	647.83 (628.73–666.94)	0.000004
Mean CVC-UR	90.32% (88.38–92.27)	90.53% (88.68–92.38)	0.881
Mean CLABSI frequency	2.84 (1.85–3.82)	3.06 (2.01–4.12)	0.764
Mean CLABSI rate	2.35 (1.5–3.2)	2.24 (1.47–3.01)	0.849
Mean CLABSI density	2.6 (1.69–3.51)	2.41 (1.62–3.21)	0.761

**Table 5:** Analysis of central line-associated bloodstream infection (CLABSI) incidence and mortality in relation to COVID-19 co-infection status among patients, along with the prevalence of alert pathogens among CLABSI etiological factors during the pandemic period (2020–2021), with sample size (*n*) and percentage of selected group.

Parameter	COVID-Positive	COVID-Negative	*p*-Value
CLABSI incidence	16/264 (6.06%)	17/904 (1.88%)	0.00037
Mortality in CLABSI patients	4/16 (25%)	1/17 (5.88%)	0.22
CLABSI caused by alert pathogens	7/16 (43.75%)	10/17 (58.82%)	0.674

**Table 6:** Adherence to the central line-associated bloodstream infection (CLABSI) prevention packages during and after the pandemic; % of fulfilled criteria among performed observations, (95% confidence interval), *p*.

Prevention Criterium	Pandemic	Post-Pandemic	*p*
Hand Hygiene Before Manipulation of a CVC	99.71 (99.15–100.28)	97.02 (94.75–99.3)	0.046
Aseptic Technique of Insertion	99.71 (99.15–100.28)	94.46 (89.7–99.22)	0.057
Catheter presence necessity documentation	98.46 (97–99.92)	97.1 (95.46–98.73)	0.238
Presence of sterile dressing	98.48 (97.31–99.65)	98.82 (97.84–99.79)	0.671
Dressing in good condition	81.32 (71.2–91.44)	88.37 (84.93–91.81)	0.218
Chlorhexidine-impregnated dressing	23.51 (17.46–29.56)	46.49 (38.09–54.9)	0.0004
Administration equipment date	97.3 (95.55–99.06)	97.12 (95.63–98.62)	0.88
Total mean compliance	85.5 (79.66–91.34)	88.48 (84.3–92.67)	0.417

## Data Availability

The de-identified individual patient data underlying this study’s findings are not publicly accessible due to the sensitive nature of protected health information from ICU patients. However, researchers may request access to this data from the corresponding author with a methodologically sound proposal. Data access requires signing a formal agreement. All data is securely stored at Wroclaw Medical University. Study-related materials (text, tables, and figures) will be provided alongside the dataset. The data will be available immediately following publication and remain accessible for 5 years thereafter.

## Abbreviations

The following abbreviations are used in this manuscript:

AMR	Antimicrobial Resistance
ANOVA	Analysis of Variance
APACHE	Acute Physiology and Chronic Health Evaluation
BSI	Bloodstream Infection
CDC	Centers for Disease Control and Prevention
CI	Confidence Interval
CLABSI	Central Line-associated Bloodstream Infection
CRBSI	Catheter-Related Bloodstream Infection
CVC	Central Vascular Catheter
CVC-D	Central-Vascular-Catheter-Days
CVC-UR	Central Vascular Catheter Utilization Ratio
ECDC	European Centre for Disease Prevention and Control
ESBL	Extended-Spectrum β-Lactamase
EUCAST	European Committee on Antimicrobial Susceptibility Testing
GRE	Glycopeptide-Resistant Enterococci
HAI	Hospital Acquired Infection
ICU	Intensive Care Unit
IQR	Interquartile Range
LOS	Length of Stay
MBL	Metallo-β-Lactamase
MDR	Multidrug-Resistant
MRSA	Methicillin-Resistant *Staphylococcus Aureus*
MRSE	Methicillin-Resistant *Staphylococcus Epidermidis*
MSSA	Methicillin-Susceptible *Staphylococcus Aureus*
MSSE	Methicillin-Susceptible *Staphylococcus Epidermidis*
NDM	New Delhi Metallo-β-Lactamase
NHSN	National Healthcare Safety Network
Pt-D	Patient-Days
SCNMR	*Staphylococcus* Coagulase-Negative Methicillin-Resistant
SD	Standard Deviation
VIM	Verona Integron-Encoded Metallo-β-Lactamase
WHO	World Health Organization
XDR	Extensively Drug-Resistant
